# Sex differences and trends in managing cardiovascular risk factors in primary care: a dynamic cohort study

**DOI:** 10.3399/BJGPO.2024.0175

**Published:** 2025-02-12

**Authors:** Geert Smits, Michiel L Bots, Monika Hollander, Sander van Doorn

**Affiliations:** 1 Julius Center for Health Sciences and Primary Care, University Medical Center Utrecht, Utrecht University, Utrecht, The Netherlands; 2 Primary Care Group PoZoB, Bolwerk, Veldhoven, The Netherlands

**Keywords:** primary health care, cardiovascular risk management, sex differences, prevention

## Abstract

**Background:**

Treatment targets for cardiovascular risk management (CVRM) make no distinction between women and men.

**Aim:**

To explore sex differences in achieving treatment targets in patients who participated in a nurse-led, integrated CVRM care programme in primary care between 2013 and 2019.

**Design & setting:**

We conducted a dynamic cohort study in the Eindhoven region, which is the south-eastern part of the Netherlands.

**Method:**

We assessed outcomes of three biological risk factors (systolic blood pressure [SBP], low-density lipoprotein [LDL] cholesterol, and estimated glomerular filtration rate [eGFR]) and four lifestyle factors (smoking, physical activity, alcohol intake, and body mass index [BMI]). Points (1 = on target; 0 = not on target) were assigned for biological risk factors, lifestyle factors, and an overall score. Using the annual results, we applied multivariable regression models to study trends over time and differences in trends between women and men.

**Results:**

The number of participants increased from 24,889 to 38,067, mean age increased from 67.3 years to 71.5 years, with around 52 % women each year. The average of seven risk factors on target increased significantly from 4.6 to 4.9 in women, and from 4.7 to 5.0 in men, with no statistical difference between women and men. Differences between women and men in 2013 in the number of both biological and lifestyle factors on target did not materially change over time.

**Conclusion:**

Integrated cardiovascular management care led to improvements in cardiovascular risk factors on target, equally well in women than in men. Differences in risk factors on target between women and men in 2013 were still present in 2019.

## How this fits in

Guidelines for cardiovascular risk management (CVRM) make no distinction in treatment targets between women and men, except for responsible alcohol intake. Between 2013 and 2019, improvements in being on target for seven modifiable risk factors was similar for women as for men who participated in a nurse-led, integrated CVRM programme. The existing difference between women and men in 2013 on target for biological risk factors (systolic blood pressure [SBP], low-density lipoprotein [LDL] cholesterol, estimated glomerular filtration rate [eGFR]) did not change over time, whereas the difference for being on target regarding lifestyle risk factors (alcohol, non-smoking, healthy exercise, body mass index [BMI]) became smaller.

## Introduction

Cardiovascular disease (CVD), in particular ischaemic heart disease (IHD) and stroke, is a leading cause of morbidity and mortality worldwide with a large societal impact in terms of life years lost and reduced quality of life.^1^ Treatment of underlying, modifiable risk factors, such as unhealthy diet, insufficient physical inactivity, tobacco use, being overweight, obesity, harmful use of alcohol, raised blood pressure, raised blood glucose, unfavourable blood lipids, and impaired renal function, can reduce the risk of myocardial infarction and stroke.^2,3^ Large-scale treatment of these modifiable risk factors in individuals can help reduce the risk at population level.

In the Netherlands, nearly all residents are registered with a GP, who has a long-term relationship with their patients and is well aware of the medical history. The GP therefore is the key healthcare provider to initiate, coordinate, and provide long-term follow-up for CVD prevention.^4^ Since 2010, GPs are increasingly organised within primary care groups that offer nurse-led, integrated cardiovascular risk management (CVRM), based on national and international guidelines, to patients eligible for participation in the CVRM care programme. In 2022, around 85% of all Dutch GPs were affiliated to a primary care group offering nurse-led, integrated CVRM care to 1.2 million patients, around 400 000 with and 800 000 without an established CVD. The CVRM care programme is carried out by a practice nurse (PN) supervised by the GP. The PN guides and supports eligible patients with adjustment of medication, adherence, and lifestyle improvements. Although the CVRM guidelines do not distinguish between women and men regarding risk factor management and treatment targets, several cross-sectional studies pointed towards differences in assessment of risk factor information^5^ and achieving treatment targets between women and men.^6–9^

Long-term longitudinal studies on this topic are lacking, despite that increased awareness on sex differences in long-term CVRM care may further improve cardiovascular health in the population.

We set out to explore trends in being on target for three biological risk factors (systolic blood pressure [SBP], low-density lipoprotein [LDL] cholesterol, and estimated glomerular filtration ratio [eGFR]) and four lifestyle factors (smoking status, daily degree of [self-reported] exercise, overweight and [self-reported] daily alcohol intake) in women and men with and without an established CVD receiving nurse-led, integrated CVRM in Dutch primary care between 2013 and 2019.

## Method

### Setting

The study was performed in the region Eindhoven, which is the south-eastern part of the Netherlands. The PoZoB primary care group comprised 137 affiliated general practices in 2013 with 415 000 registered patients, covering rural, suburban, and urban practices and can be considered as representative for the situation in the Netherlands. Identification of patients eligible for the CVRM care programme occurred stepwise between 2010 and 2013, and has been described in detail previously.^10,11^

### Study population

Patients eligible for the integrated CVRM care programme visited the PN 1–4 times a year. Conditions for eligibility were based on the 2012 CVRM guidelines of the Dutch College of General Practitioners.^12^ These conditions were similar for all new patients who entered the CVRM care programme until 2019. The inclusion and exclusion criteria for the eligibility of integrated CVRM care are described in Table 1. A distinction was made between patients with and without an established CVD. At least once a year a history was taken by the PN regarding medication adherence and lifestyle adjustments, followed by a protocolised physical examination to assess blood pressure, pulse frequency, height and weight to calculate body mass index (BMI). Furthermore, blood and urine tests were taken for measurement of fasting glucose, serum lipids, renal function (expressed as eGFR and albuminuria). Data were collected in the care group’s multidisciplinary information system (Care2U), a separate database for the chronic care programmes. Data in Care2U were automatically transferred to the patient’s electronic health record (EHR).

**Table 1.** Inclusion and exclusion criteria for participation in the CVRM care programme

Inclusion criteria for patients with an established CVD:
Patients with a documented atherosclerotic CVD, including angina pectoris, myocardial infarction, chronic ischaemic heart disease, coronary sclerosis, TIA, cerebral infarction, intermittent claudication or aneurysm of the abdominal aorta AND The patient is primarily managed by the GP AND Aged ≥18 years
Inclusion criteria for patients without an established CVD:
No previous CVD AND Use of antihypertensive or lipid-lowering drugs OR A 10-year CVD morbidity and mortality risk >10%, based on the Dutch guideline for CVRM^4^ OR A SBP >180 mm Hg and/or a TC/HDL-ratio >8, regardless of the 10-year CVD risk AND The patient is primarily managed by the GP AND Aged ≥18 years
Exclusion criteria for all patients:
DM, as these patients receive CVRM in a DM programme Patient receives CVRM care in the hospital or outpatient clinic from a medical specialist

CVD = cardiovasular disease. CVRM = cardiovascular risk management. DM = diabetes mellitus. SBP = systolic blood pressure. TIA = transient ischaemic attack. TC/HDL = total cholesterol-to-high-density lipoprotein

### Risk factor targets

Risk factor targets were derived from the Dutch guideline for CVRM 2012:^4^ (i) biological risk factors: SBP ≤140 mmHg, LDL cholesterol ≤2,5 mmol/l, eGFR ≥60 ml/min/1.73m^2^; (ii) lifestyle-related risk factors: body mass index (BMI) ≤25 kg/m^2^, sufficient physical exercise (according to the Dutch norm for healthy exercise: 5 days a week at least 30 minutes moderate exercise per day), non-smoking, and the number of alcoholic units per day (≤1 for women, ≤2 for men). Points were assigned for risk factor control, where a risk factor on target was 1 point, and not on target was 0 points. Thus, three and four points for biological and lifestyle risk factors respectively, could make up to a total score of seven points.

### Statistical analyses

First, the dataset comprised seven cross-sectional datasets (2013–2019). Each year was restricted to those individuals who had information on all seven targets, and so the analyses are considered a complete case analysis. General characteristics of the studied population are presented as means and standard deviation (SD) for continuous variables and as percentages for categorical variables for each year. Second, annual mean number of the risk factors on target are presented graphically by year with corresponding 99% confidence limits, for women and men, and in strata of previous cardiovascular history. Next, multivariable linear regression models were applied to obtain the slope (change per year) in the number of factors lifestyle on target, risk factors on target, and all seven modifiable factors on target. Slopes were presented with 99% confidence intervals (CIs). Results were presented for women and men separately, and additionally in strata of individuals with and without a previous cardiovascular history. Finally, annual sex-specific graphs are presented in a supplement to show the percentage of each risk factor being on target in the population. Statistical analyses were performed with SPSS statistical software (version 27).

## Results

General characteristics as annual cross-sectional datasets of the eligible population for the period 2013 up to 2019 are presented in Table 2 (women) and in Table 3 (men). In women, we saw an increase of patients on target for LDL cholesterol (2013: 28%, 2019: 49%), SBP (2013: 66%, 2019: 70%) and eGFR (2013: 79%, 2019: 84%), BMI (2013: 34%, 2019: 35%), responsible alcohol consumption (2013: 87%, 2019: 89%), and non-smoking (2013: 87%, 2019: 88%), and a decrease in sufficient physical activity (2013: 80%, 2019: 75%).

**Table 2.** General characteristics of the studied PoZoB population for women (*n* = 118 409), presented by calendar year

	2013	2014	2015	2016	2017	2018	2019
Number of women	13 269	15 234	17 126	17 215	17 457	18 688	19 420
Age in years (mean, SD)	69.4 (10)	69.9 (10)	70.3 (10)	70.5 (10)	70.8 (10)	71.2 (10)	71.5 (10)
% enrolled for secondary prevention	30	32	33	35	38	38	39
% LDL ≤2.5 mmol/l	28.1	34.1	42.9	42.2	44.3	57.2	48.7
% SBP ≤140 mmHg	66.2	66.6	67.5	68.8	69.7	70.0	70.2
% eGFR >60 ml/min/1.73 m^2^	79.4	83.7	87.1	84.1	82.9	83.7	84.1
% BMI ≤25 kg/m^2^	33.8	33.8	34.3	34.3	34.1	34.3	34.7
% currently non-smoking	86.5	86.7	86.9	86.8	87.3	87.5	87.9
% sufficiently physical active	80.2	76.9	75.9	75.0	75.2	75.1	75.0
% on guideline-based alcohol consumption	86.8	86.8	87.1	87.7	87.8	88.1	88.7
% on blood pressure-lowering medication	65.3	63.3	64.7	63.6	68.7	70.4	74.5
% on lipid-modifying medication	42.1	43.3	47.4	47.9	53.0	53.1	55.2
Number of lifestyle factors on target (max 4) (mean, SD)	2.9 (0.2)	2.8 (0.2)	2.8 (0.2)	2.8 (0.2)	2.8 (0.2)	2.9 (0.2)	2.9 (0.2)
Number of biological risk factors on target (max 3) (mean, SD)	1.7 (0.3)	1.8 (0.3)	2.0 (0.3)	2.0 (0.3)	2.0 (0.3)	2.1 (0.3)	2.0 (0.3)
Number of all modifiable factors on target (max 7) (mean, SD)	4.6 (0.7)	4.7 (0.7)	4.8 (0.7)	4.8 (0.7)	4.8 (0.7)	5.0 (0.7)	4.9 (0.7)

BMI = body mass index. eGFR = estimated glomerular filtration rate. LDL = low-density lipoprotein. SBP = systolic blood pressure. SD = standard deviation

**Table 3.** General characteristics of the studied PoZoB population for men (*n* = 107 879), presented by calendar year

	2013	2014	2015	2016	2017	2018	2019
Number of men	11 620	13 342	15 175	15 423	16 123	17 549	18 647
Age in years (mean, SD)	67.0 (9.5)	67.6 (9.7)	67.9 (9.8)	68.3 (9.9)	68.6 (10)	69.1 (10)	69.4 (10)
% enrolled for secondary prevention	40	41	43	45	48	50	50
% LDL ≤2.5 mmol/l	35.9	41.6	49.5	49.3	52.9	66.1	57.7
% SBP ≤140 mmHg	67.2	68.5	68.8	70.3	71.9	72.5	71.7
% eGFR >60 ml/min/1.73 m^2^	87.2	89.5	91.5	88.5	87.5	88.3	88.3
% BMI ≤25 kg/m^2^	26.2	26.5	26.6	26.8	26.9	27.4	27.5
% currently non-smoking	83.3	84.3	85.0	85.3	86.0	86.5	87.2
% sufficiently physical active	83.2	80.4	79.6	78.9	78.9	79.4	79.6
% on guideline-based alcohol consumption	87.1	87.4	87.7	88.2	88.9	89.3	90.1
% on blood pressure-lowering medication	62.6	60.9	62.8	62.2	67.8	69.0	73.2
% on lipid-modifying medication	48.0	48.7	53.0	54.8	61.2	61.1	63.9
Number of lifestyle factors on target (max 4) (mean, SD)	2.8 (0.8)	2.8 (0.8)	2.8 (0.8)	2.8 (0.8)	2.8 (0.8)	2.8 (0.8)	2.8 (0.8)
Number of biological risk factors on target (max 3) (mean, SD)	1.9 (0.8)	2.0 (0.7)	2.1 (0.7)	2.1 (0.8)	2.1 (0.8)	2.3 (0.7)	2.2 (0.8)
Number of all modifiable factors on target (max 7) (mean, SD)	4.7 (1.1)	4.8 (1.1)	4.9 (1.1)	4.9 (1.1)	4.9 (1.1)	5.1 (1.1)	5.0 (1.1)

BMI = body mass index. eGFR = estimated glomerular filtration rate. LDL = low-density lipoprotein. SBP = systolic blood pressure. SD = standard deviation

In men we saw an increase of patients on target for LDL cholesterol (2013: 36%, 2019: 58%), SBP (2013: 67%, 2019: 72%), eGFR (2013: 87%, 2019: 88%), BMI (2013: 26%, 2019: 28%), responsible alcohol consumption (2013: 87%, 2019: 90%), non-smoking (2013: 83%, 2019: 87%), and a decrease in sufficient physical activity (2013: 83%, 2019: 80%).

**Figure 1.**
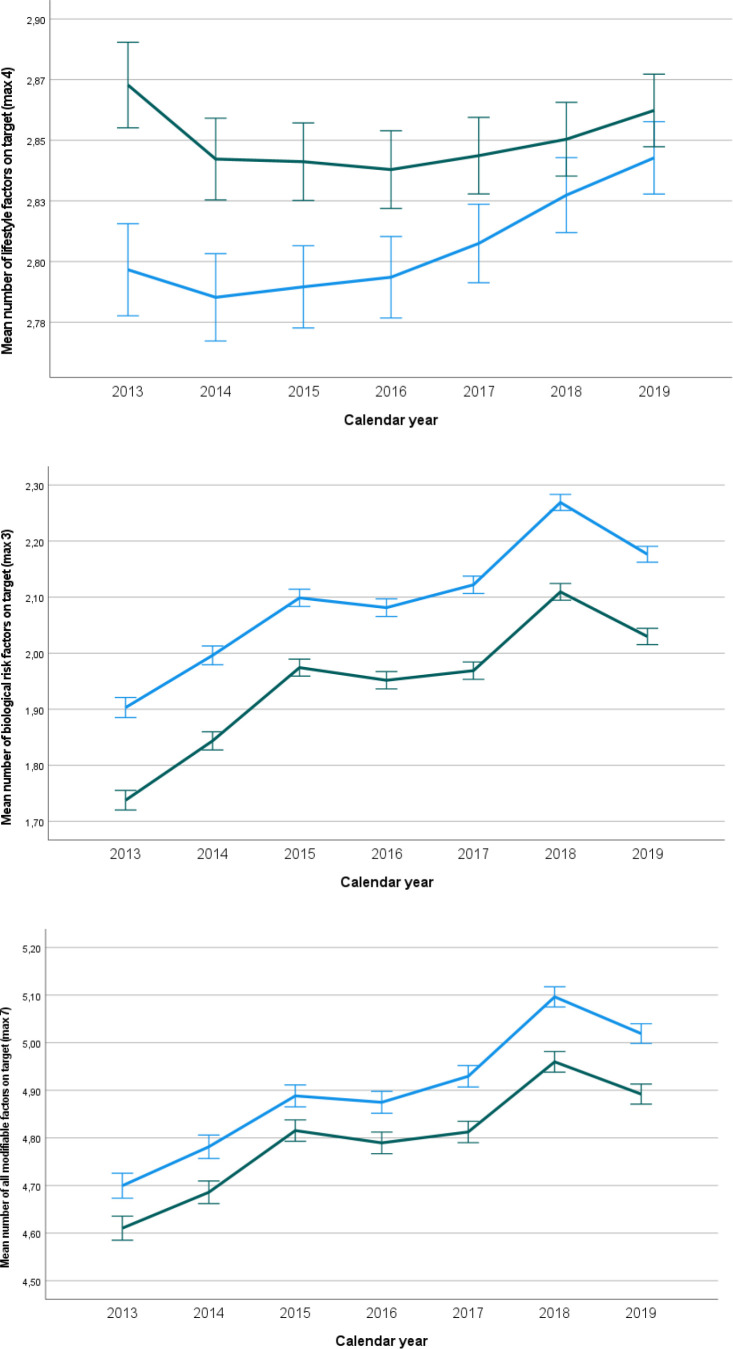
Mean number of factors on target with 99% confidence limits between women and men over time for the entire population (unadjusted results). From top to bottom: lifestyle risk factors (*n* = 4), biological risk factors (*n* = 3), all factors (*n* = 7). Green line: women, blue line: men.

**Figure 2. fig2:**
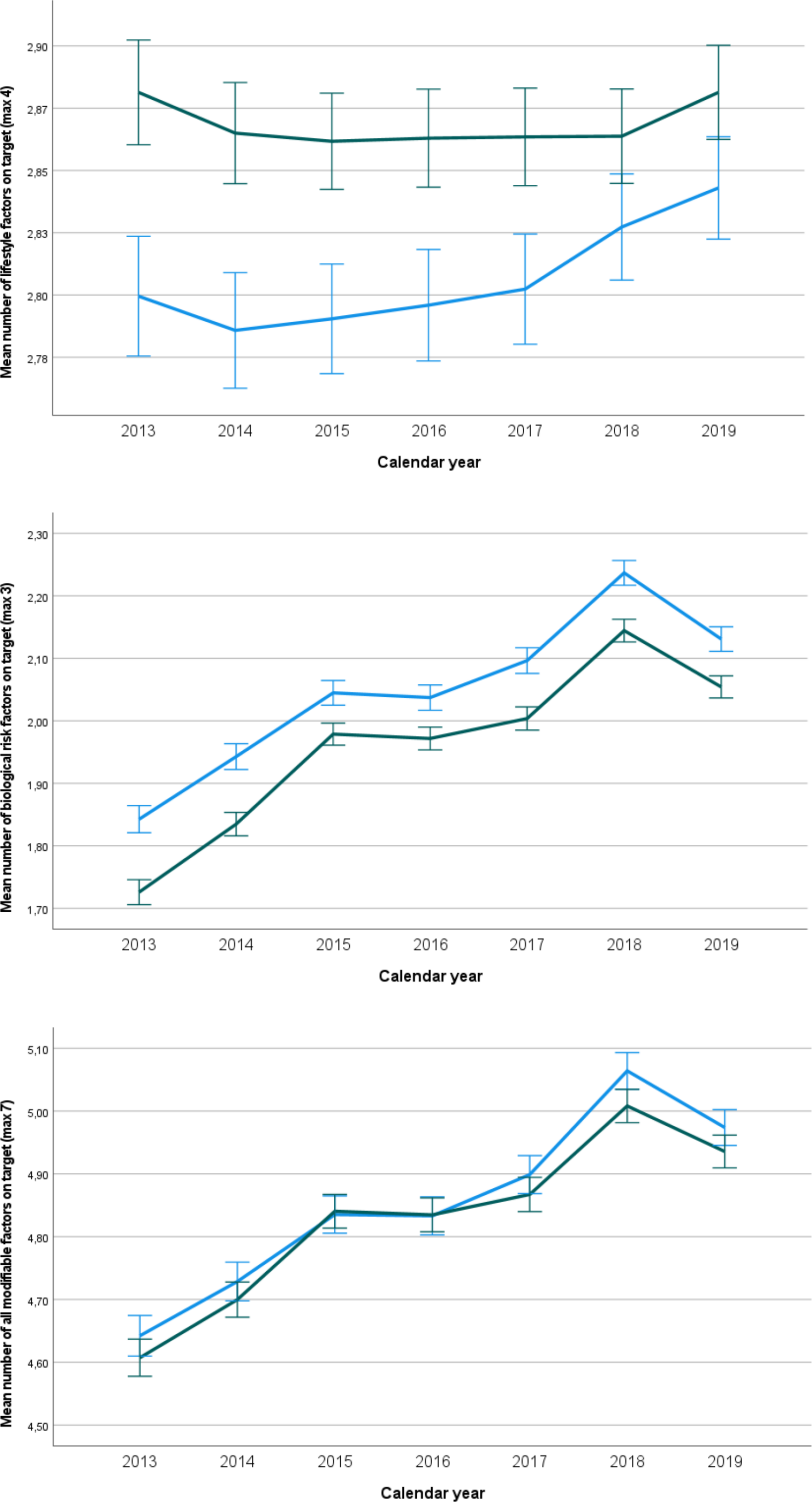
Mean number of factors on target with 99% confidence limits between women and men over time for the population without an established cardiovascular disease (unadjusted results). From top to bottom: lifestyle risk factors (*n* = 4), biological risk factors (*n* = 3), all factors (*n* = 7). Green line: women, blue line: men.

**Figure 3.**
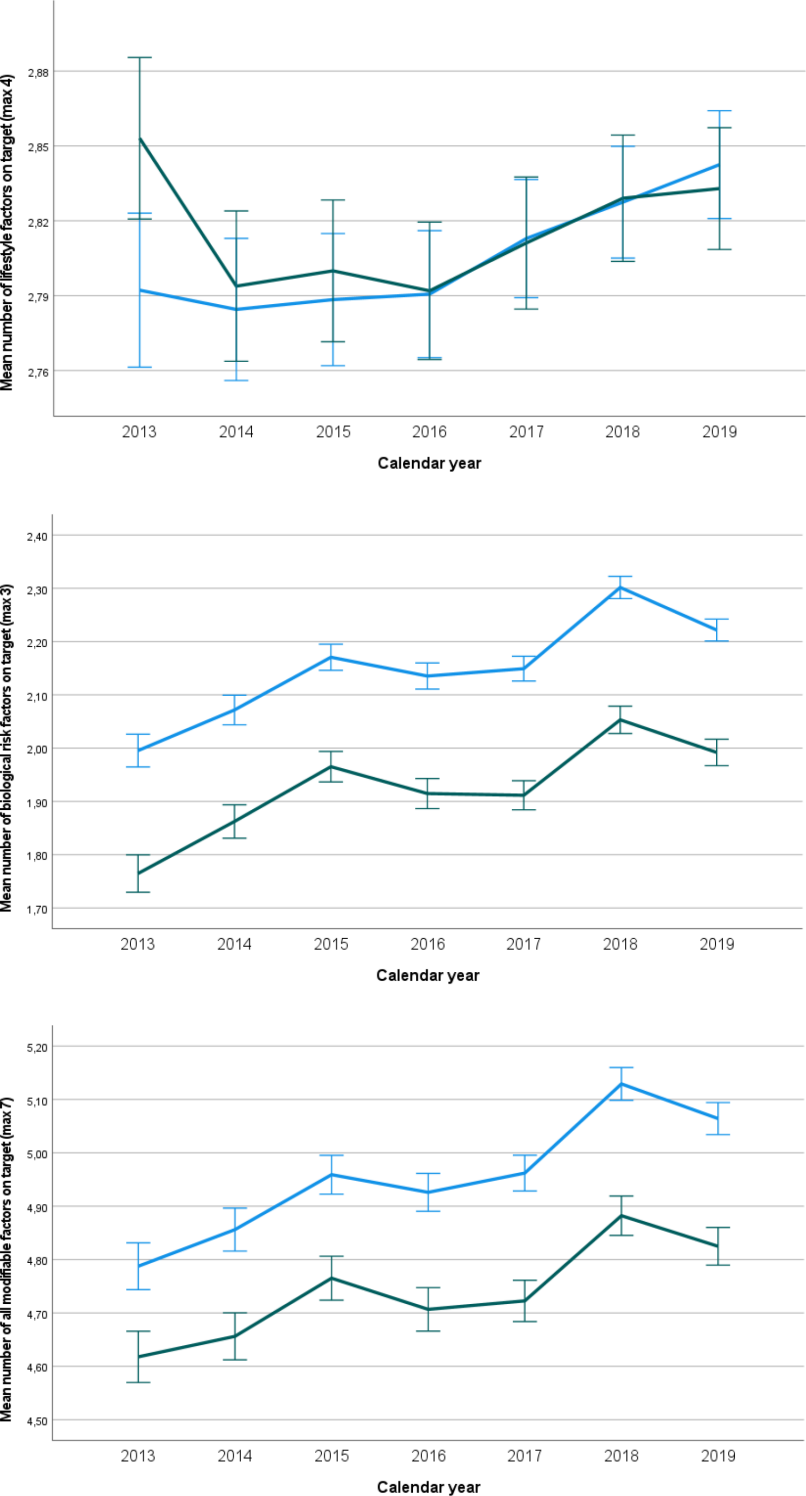
Mean number of factors on target with 99% confidence limits between women and men over time for the population with an established cardiovascular disease (unadjusted results). From top to bottom: lifestyle risk factors (*n* = 4), biological risk factors (*n* = 3), all factors (*n* = 7). Green line: women, blue line: men.

If we look at the changes over time (Table 4) in the total population, we see that the number of seven modifiable factors being on target increased with 0.054 per year (99% CI = 0.050 to 0.058) in women and with 0.055 per year (99% CI = 0.051 to 0.060) in men. Similar relations were found for the number of biological risk factors on target. Only for lifestyle factors, improvement was more pronounced in men than in women, without reaching statistical significance. In individuals with and without an established CVD, we see similar results in terms of direction and magnitude. In Figures 1 [Fig fig2]-3, the mean numbers on target for lifestyle riskfactors (3), biological riskfactors (4) and the total number of riskfactors (7) are given for the entire population, for the poulation without an established CVD and for the population with an established CVD , respectively.

**Table 4.** The relation between calendar year and number of modifiable risk factors on target, for men and women overall and in patients with and without an established CVD (b)

	Men		Women	
	Estimated slope (per year) (a)	99% Confidence limit	Estimated slope (per year) (a)	99% Confidence limit
Lifestyle score	0.006	0.003 to 0.009	0.0	0.030 to -0.030
Biological risk factor score	0.049	0.046 to 0.052	0.054	0.051 to 0.057
All 7 factor score	0.055	0.051 to 0.060	0.054	0.050 to 0.058
**Patients with an established CVD**				
Lifestyle score	0.006	0.001 to 0.011	0.020	-0.040 to 0.070
Biological risk factor score	0.044	0.040 to 0.049	0.045	0.040 to 0.050
All 7 factor score	0.050	0.044 to 0.057	0.047	0.039 to 0.054
**Patients without an established CVD**				
Lifestyle score	0.006	0.002 to 0.010	-0.010	-0.040 to 0.030
Biological risk factor score	0.054	0.050 to 0.058	0.059	0.056 to 0.063
All 7 factor score	0.060	0.054 to 0.066	0.059	0.054 to 0.064

^a^Estimates reflect the change in the outcome (number of factors on target) per calendar year increase. The relation was assessed using multivariable linear regression models adjusted for age and CVD history, when appropriate. We provide 99% confidence limits (corresponding to a *P*-value of 0.01). So when the 0 is not in the confidence limits, the statistical significance *P*-value <0.01. ^b^When the 99% confidence intervals of the estimated slopes of those for women with those for men do not overlap, is an conservative approach to indicate statistical significance with a *P*-value <0.01. CVD = cardiovascular disease

In the supplementary files information on sex differences and trends by year in percentage of the population that is on target is presented for each of the risk factors separately (Supplementary Information S1–S7) and for the percentage of patients on blood pressure-lowering (BPL) and lipid-modifying (LM) medication (Supplementary Information S8 and S9). Regarding individual risk factors, the mean levels of achieved targets and trends over time did not differ between women and men for BMI, LDL cholesterol, SBP, and eGFR. However, for the risk factor 'non-smoking', the gap between women and in men became smaller, because the improvements observed were stronger in men than in women. The gap widened for the risk factors 'physical activity' and 'responsible alcohol consumption', because of slightly stronger improvements in men (Supplementary Information S1–S7). Furthermore, the gap between women and men on BPL medication slightly seems to narrow (Supplementary Information S8), but the gap between women and men on LM medication is growing (Supplementary Information S9).

## Discussion

### Summary

In this study, we evaluated sex differences and trends in the management of cardiovascular risk factors of patients who participated in a nurse-led, integrated CVRM care programme between 2013 and 2019. Women with a history of CVD did worse than men in both biological and lifestyle risk factors, women without a history of CVD did worse than men in biological, but better in lifestyle risk factors. In most risk factors, differences between men and women were small except for BMI. Sex differences observed at the start of the study remained present until 2019, with hardly any changes in the magnitude of the differences.

### Strengths and limitations

A major strength is that we able to visualise a long-term trend in reaching CVRM targets in a large cohort of patients who participated in an integrated CVRM care programme. Second, eligibility for the nurse-led CVRM care programme was based on criteria from the national CVRM guideline,^12^ which increases the external validity (that is, generalisability). Third, with three biological and four lifestyle risk factors it was possible to present a fairly complete picture of CVRM in high-risk patients with and without previous CVD. Fourth, the percentage of missing data for the seven risk factors was only 10% annually, owing to data collection by trained PNs in the care groups’ multidisciplinary information system (Care2U). There are also a number of limitations. First, we have no information on caregivers and patients’ arguments for not starting or increasing medication or lifestyle adjustments. Second, some lifestyle risk factors (alcohol intake, physical activity) were self-reported and differences on reporting of these risk factors may exist. Third, we did not have data on dietary habits, another important lifestyle risk factor.

### Comparison with existing literature

To our knowledge, the Netherlands is the only country that offers widespread, continuous, long-term, nurse-led integrated CVRM care to high-risk patients with and without an established CVD. Health survey data from the UK and the US on cardiovascular risk factors show decreasing trends in patients with hypertension, hypercholesterolemia, and smoking, with women better than men in control for hypertension and non-smoking, but worse than men for hypercholesterolemia.^13,14^ Both studies showed a decrease of patients with their BMI on target, affecting more women than men, while in our study the BMI remained unchanged over time with women more often on target for BMI than men. In our study, we found that women were more likely to be non-smoking, but the gap between women and men was narrowing. It is known that women have more problems achieving long-term tobacco abstinence; however, reasons for this are unclear.^15^ Regarding physical activity, we found conflicting results. The decrease in sufficient physical activity in our study was confirmed in a study of Kotseva *et al* who saw no increase in physical activity in patients with coronary conditions between 1999 and 2013,^16^ while a minimal increase in the prevalence rate of physical activity between 2007 and 2018 was observed in the US.^17^

Although in our study improvements were seen for both sexes between 2013 and 2019 in six out of seven risk factors, management in 2019 was still suboptimal with 49 %, 70 %, and 84 % of the women on target for LDL cholesterol, SBP, and eGFR, respectively, and 58 %, 72 %, and 88 % of the men on target, respectively. Control of SBP was a little lower in women compared with men (70 % versus 72 %), although women were slightly more often prescribed BPL medication than men (75 % versus 73 %). LM medication was more often prescribed to men (2013: 42 % versus 48 %) over time and the gap seems to have only increased in subsequent years (2019: 55 % versus 64 %). Sex differences in prescribing LM medication may be explained by the fact that women were 2 years older than men (2013: 69.4 years versus 67.0 years in 2013 and 71.5 years versus 69.4 years in 2019), which might have translated in more comorbidity, more comedication, and more reluctance to increase LM medication, both by the patient as well as the PN or the GP.^18^ Furthermore, it is known that women are more worried about safety and side effects of statins and are more willing to try natural remedies, such as diet and exercise, to lower their LDL cholesterol.^19,20^ Many studies on sex differences in primary care with a cross-sectional design reported that women less likely had their LDL cholesterol on target^5,21–24^ and more likely had their SBP on target than men.^5,22–24^

### Implications for research and practice

To reduce sex differences in biological risk factor control, primary health caregivers need to explore reasons for not prescribing or increasing medication, especially in women. An individual care plan, in which the lifestyle adjustments chosen by the patient are registered, may contribute to improved exercise, changing dietary habits, and weight loss.

In conclusion, pharmacological treatment and control in women should receive more attention to improve outcomes of biological risk factors.

## Data

Data for this article were provided by the PoZoB primary care group and JGPN. Data will be shared on request to the corresponding author with permission of the care groups’ board and with permission of the JGPNs board.
